# Improving regulatory efficiency for biotechnology products

**DOI:** 10.3389/fbioe.2024.1354743

**Published:** 2024-01-18

**Authors:** Muffy Koch, Matthew G. Pence, Jaylee DeMond, Gary Rudgers

**Affiliations:** Simplot Plant Sciences, J. R. Simplot Company, Boise, ID, United States

**Keywords:** regulation, risk assessment, decision making, data requirements, regulatory efficiency

## Abstract

Small procedural changes in how regulatory agencies implement biotech policies can make significant differences in improving regulatory efficiency. This paper discusses how science based, crop specific guidance documents can improve dossier content and the review and approval of biotech varieties. In addition, we describe how the adoption of established risk assessment methodology and applying policy-linked decision making at the agency level can boost both efficiency and developer, public and government confidence in agency decision making and in biotech crops.

## Introduction

Biotech plant varieties have the potential to offer society more sustainable food production, improved food security, and more appealing foods. However, before these benefits can reach farmers, markets, and consumers, they rely on an efficient, predictable, and timely regulatory review process. In addition, national decisions on biotech safety evaluations can build consumer confidence when they are linked to national policies that provide clarity on why biotech products are important for the country (Raybould, 2021).

Many improvements in the efficiency of regulatory review processes do not require changes to regulations or rulemaking. Based on a decade of experience obtaining approvals for food safety and environmental release of more than ten biotech potato varieties in twelve countries, we have noted recurring gaps in some national regulatory procedures. These gaps significantly slow down the review, approval, and marketing of biotech products. In this paper we focus on three of these gaps: the absence of crop specific data requirements, poor risk assessment methodology, and delayed decision making. We show that these regulatory delays impact on farmer access to improved planting material, the financial cost of regulation, and consumer acceptance, and we suggest ways to improve regulatory efficiency and decision making without compromising food or environmental safety reviews.

## Crop-specific data requirements

To keep regulatory agencies effective and efficient, it is important that data requirements for safety reviews are written in guidelines, not in regulations or Acts. For the last 30 years, agencies have reviewed hundreds of new biotech varieties, many of which are seed crops like cotton, maize, and soybean (ISAAA, 2019). Early biotech products helped to formulate the regulatory procedures and requirements in many countries. In some countries, general data requirements for safety assessments that are appropriate for these seed crops, are written into the laws (Acts) that govern release and use of biotech products. Data requirements in these laws make it difficult or impossible for regulators to adapt their review processes to new or different crops where seed crop-specific data requirements are inappropriate.

**FIGURE 1:**
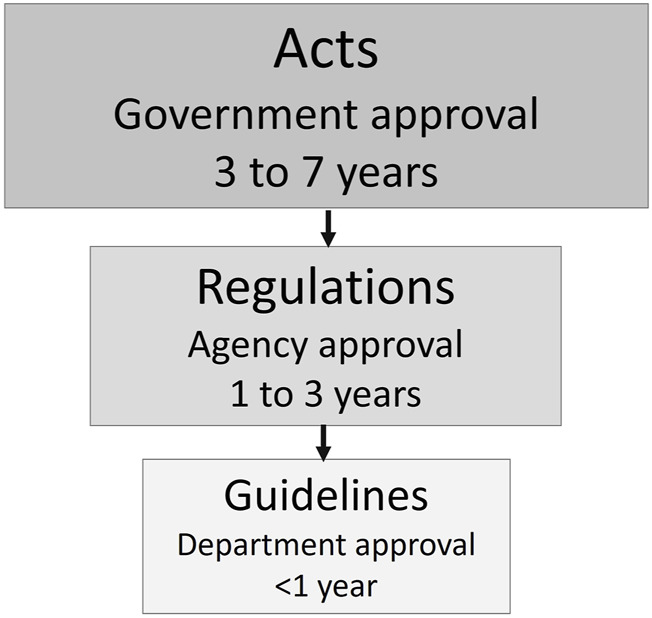
Hierarchy of legal instruments and time to implementation.

Generally, Acts are overarching legal instruments that govern broad categories of activities and things, such as biosafety laws that regulate biotechnology and the products of biotechnology (Figure 1). Changing or establishing an Act can take many years depending on the country and political priorities. Regulations are developed under the authority of Acts; they lay out processes for implementation and may narrow the scope of implementation to specific high-risk activities or products. Updating regulations can take several years and generally requires public consultation and interagency review. Guidelines, developed by the implementing department, help provide clarity and navigate stakeholders through the regulatory process. Guidelines are working documents that can be developed more easily and are focused on specific processes that need clarification. They can be updated and edited as regulated activities, new technologies, and science evolve.

Guidelines are most useful when they are crop specific, published proactively, and developed in consultation with stakeholders. Good examples of crop specific guidelines for biotech crops are provided by the Canadian Food Inspection Agency (CFIA; Crop-specific terms and conditions—Canadian Food Inspection Agency (canada.ca)). This agency proactively develops crop-specific guidelines when they are informed that new biotech varieties will be entering the Canadian regulatory system. These guidelines give developers a clear understanding of the terms and conditions that will apply to the design and running of their field trials and improve the developer’s ability to provide science-based, well-structured applications to the agency. Similarly, CFIA develops biology guidance documents for specific crops to help developers prepare for environmental safety assessments when the varieties move to regulatory approval for commercial use (Biology Documents—Companion Documents for Directive 94-08—Canadian Food Inspection Agency (canada.ca)).

Simplot has been working with biotechnology to improve potatoes for the past 20 years. In that time there have been requests from regulators for data on analytes that are important in seed crops but are not present at measurable levels in potatoes, genetic stability data for a clonally propagated crop, and grain seed handling standards applied to perishable potato seed tubers.

In our experience, composition analysis of biotech crops runs into the tens of thousands of United States dollars (US$) for each biotech product. Analytes such as lipids and phytase levels are important composition requirements for some crops, such as cotton, canola, and soybean, but these analytes are typically below the level of detection in potato tubers. One way to improve efficiency of the regulatory review process would be to remove data requirements from laws or regulations where they require biotech developers to collect data that is not relevant to safety reviews and can be costly. AgBioInvestor (2022) calculated the average cost to bring a biotech event to the market was US$ 115 million and of this, US$ 43.2 million was spent on regulatory compliance and approval, 37% of the total expense. These costs would be prohibitive for public sector and small to medium enterprises trying to bring improved biotech varieties to the market. In addition, amending regulations for each crop is often impractical and time consuming. However, countries that provide crop specific data requirements in guidance documents can rapidly adapt the requirements based on the crop under review.

Similarly, genetic stability is important for inheritance and breeding in seed crops but has no impact during the clonal propagation of vegetative crops like potatoes, sweet potatoes, bananas, strawberries, and ginger. Because the suggestion to check stability comes from international risk assessment guidance (CODEX CAC/GL 45-2008), some agencies feel obligated to request these data for vegetative crops even though clonal propagation does not involve the major contributors to genetic instability, i.e., meiosis, segregation, or recombination (Pence et al., in this publication). Genetic stability data requirements have no scientific justification for vegetative crops and can be costly, which adds regulatory burden to all developers, including small developers and the public sector. Agencies could use science-based decision making to remove data requirements, such as stability data for vegetative crops, that provide no input on safety of the crop under review.

Some countries have a regulatory requirement that the biotech variety being reviewed must be grown and tested locally in their country for safety assessment, even when the application is specifically for food safety approval for imported food products. This additional testing is duplicative based on identical studies already conducted in the country of origin and is often required even when the crop will not be cultivated in that country either due to environmental conditions or phytosanitary regulations prohibiting the importation or cultivation of the plant. When regulations require the developer to provide planting material, the regulators request that seed is shipped to the agency for field testing. This process is suitable for true seed, such as corn or soybean, which stores well for months at room temperature, but is not feasible for vegetative propagation material, like potato seed tubers, which are bulky and perishable. Shipments of vegetative planting material require specialized transport, equipment, and sufficient storage space in controlled environments, which are rarely available at regulatory offices. In addition, importation of potato tubers is often restricted or prohibited by phytosanitary regulations.

Unless regulatory requirements are appropriate for different crops, they delay the regulatory process and remain a problem for developers. Other mechanisms to ensure that required safety data are appropriate for specific crops include enabling pre-submission consultations with regulators and enabling the use of data waivers where it is agreed that current data requirements are not suitable for specific products. Pre-submission consultations allow developers to discuss data requirements with the regulators and to request data waivers where there is justification for this allowance.

In addition, there is evidence supporting the transportability of risk assessment data between countries and agroclimatic zones (Nakai et al., 2015; Vesprini et al., 2020; Backman et al., 2021). Laboratory, greenhouse, and field data generated for a biotech crop in one country can be used to support risk assessment for the same crop in other countries (Bachman et al., 2021). This practice is implemented by regulatory agencies in countries like Argentina, Brazil, Canada, and Japan, for environmental risk assessment (Nakai et al., 2015; Vesprini et al., 2020) and in most countries for food safety risk assessments. Policy decisions that enable agencies to consider data generated in other countries for environmental safety and food safety could improve efficiency of regulatory reviews for new biotech products. Using this approach, only the identification of a potential hazard specific to the country and not yet adequately addressed by existing data would require additional assessment and might require country specific data (Bachman, et al., 2021).

## Risk assessment methodology

Risk assessment methodology is well established and many guides exist for its application to biotech products, for example, the Australian Office of the Gene Technology Regulator’s Risk Analysis Framework (OGTR, 2013). The risk assessment methodology applied in biotech regulatory agencies varies widely from country to country. Even established agencies with years of experience sometimes appear to improperly apply established risk assessment methodology. As a result, developers receive a substantial number of questions with no identified safety concern or pathway to harm.

**FIGURE 2:**
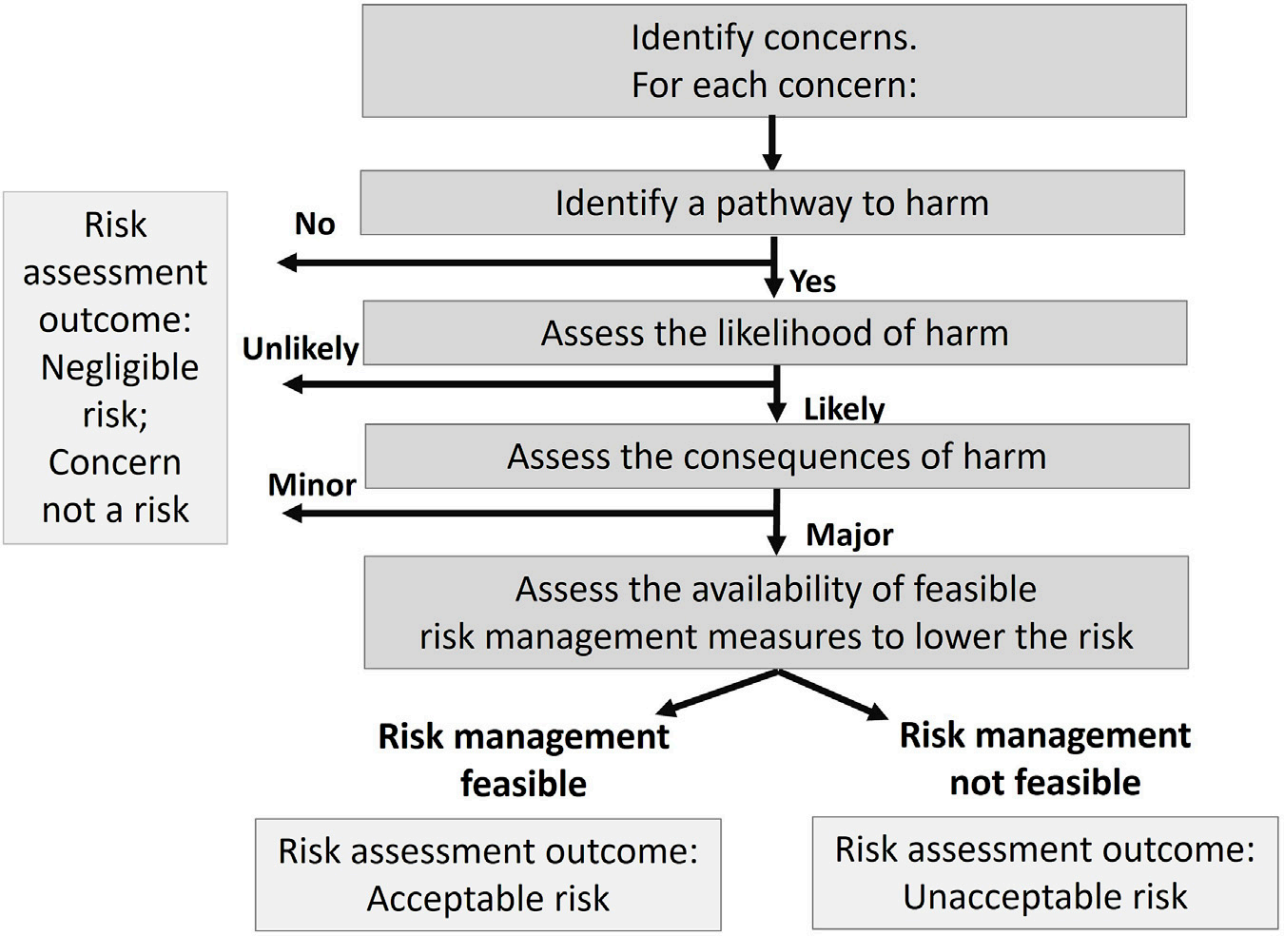
Risk assessment methodology.

Risk assessment, which includes risk identification and risk management steps, is used to identify plausible pathways to harm, assess the likelihood that harm will occur, understand the consequences should harm occur, determine whether risk management can reduce the likelihood or consequences of harm, and assess whether the risk of each safety concern is acceptable or unacceptable in the local context. This stepwise risk assessment aligns with problem formulation (Wolt, et al., 2010) and identifies and describes safety concerns and is used to categorize them into i) concerns with no plausible mechanism for harm to occur, ii) concerns with low likelihood or minor consequence, and iii) concerns with unacceptable risk that need further clarification and risk management (Figure 2). Safety concerns with no or low risk, generally do not require additional information from the developer. Concerns with plausible pathways to harm, a likelihood of harm, and potentially major consequences, become the focus of the risk assessment.

Questions from regulators to developers can be formulated to obtain information that will clarify the likelihood and consequences of risk, understand applicable risk management measures, and bring risk to an acceptable level in the local context in order to make a risk assessment conclusion that can be used to guide decision making. Documenting the risk assessment process records all the concerns that were considered and identifies potential hazards that need to be addressed before the product can be approved. A summary of this record in the final decision document provides transparency and helps to build public trust in the regulatory process.

In our experience, use of this methodology varies widely from agency to agency. In agencies where risk assessment is not fully implemented, Simplot has seen a substantial number of questions asked that have no impact on product safety and with no defined decision point. For example, Simplot has a biotech potato variety that has been approved by food safety agencies in nine countries and has been on the market in the United States for over 8 years but has yet to receive food safety approval in some countries. These delays limit commercial release of this product, however, millions of servings have been eaten without any reported safety issue. In one country, the agencies reviewing the product have asked 173 questions during the 5-year review period. Questions for nine previous approvals of this product averaged eight per country. An analysis of the 173 questions shows that 66% have no identified risk basis. In addition, while this application is specifically for food safety approval, 12% of the questions relate to environmental safety of the biotech potato when grown in the import country. Of the questions asked, 31% are duplicate questions asked by different agencies within the same country. Finally, 9% of the questions could be addressed by involvement of a local potato expert on the review committees. Many of the questions are curiosity questions raised by scientists interested in the product or the technology. In biosafety terms these would be considered ‘nice to know’ questions with no bearing on safety. Functioning risk assessment methodology would ensure that all questions reaching the developer were ‘need to know’ with direct bearing on the safety of the product. This example illustrates the regulatory barriers that developers face when regulatory processes are not efficient and are not risk-based.

Global harmonization of risk assessment and regulatory approvals is an achievable goal that would accelerate the approval and deployment of safe biotech crops and products. An example of this is the 2014 policy decision in Vietnam that any biotech product with food safety approval in five developed countries would be eligible for food safety approval in Vietnam without additional review or data requirements (Ministry of Agriculture and Rural Development, 2014). This is a science-based policy decision that makes it easier for developers to move new biotech crops to the market. Facilitated approval of biotech products in import countries ensures that farmers in production countries can benefit from the improved planting material without long delays. As more biotech products are developed by public sector scientists, who may not have the expertise and funding to complete regulatory approvals in multiple countries, regulatory efficiencies will be essential if countries are to benefit from the improvements these crops bring to the sustainability of food production.

## Policy-linked decision making

In countries where socio-economic considerations such as the impact on food prices, consumer acceptance, or farmer access to seed, are part of the decision process, the risk assessment outcome is not the final regulatory decision. However, where approvals are based on safety, the risk assessment conclusion determines the decision, and the agency quickly issues a regulatory decision based on safety of the product. These are risk-based regulatory systems and are the most supportive of new biotech variety introduction. In countries where the risk assessment is not the only factor that informs regulatory decisions, decisions can be influenced by the socio-political environment, such as pressure from groups in opposition to biotechnology or concerns that approval of biotech products will impact negatively on careers of the decision makers. For this reason, reaching a safety conclusion at an agency does not necessarily mean reaching a quick decision. An example of this is provided in Table 1 for one potato variety in 12 countries.

**TABLE 1:** Timelines for risk-based vs. socio-political food safety decision making for one biotech potato variety.

Decision Criteria	Country	Time to risk conclusion (months)^a^	Time to decision (months)^b^
Risk based	1	25	25
	2	31	31
	3	9	9
	4	52	52
	5	12	12
	6	12	12
	7	8	8
	8	8	8
	9	18	18
Socio-political	10	36	88+^c^
	11	30	93+
	12	92+	92+

^a^ Time until no more questions were received and/or agency noted the risk assessment was complete.

^b^ Time until the regulatory decision was issued.

^c^ + = decision not yet made.

There is a clear difference in the regulatory timelines between risk-based decision making and socio-political decision making (Table 1). Average time to risk conclusion with risk-based assessment was 63% faster than socio-political decision making (19.4 months vs 52.7 months). Moreover, time to decision was 79% faster when the assessment was risk based (19.4 months vs. 91 months). These delays restrict farmer access to new potato varieties in production countries and inhibit the ability of developers to release new crop varieties in these countries. Delaying farmer access to new varieties restricts their access to sustainable production benefits, thereby limiting the ability of production countries to address climate challenges.

Raybould (2021) discussed the delays and problems with biotech regulatory decision making and noted that regulatory decisions are inevitably political because decision makers decide whether particular products will help or hinder the delivery of public policy objectives. He suggested to regulators that a way to overcome the political uncertainty of biotech decision making would be to include in decisions the impact products could have on national policies, or locally adopted international policies. Many countries have policies for food security, poverty alleviation, rural development, sustainable agriculture, climate mitigation, and technology innovation that are supported by the regulatory approval and use of improved crop varieties, even those developed using biotechnology. In addition to these national policies, many countries have adopted United Nations Millennium Development Goals (United Nations, 2000) and Sustainability Goals (United Nations, 2015). The Millennium Development Goal #1 is to eradicate extreme poverty and hunger, and the Millennium Development Goal #7 is to ensure environmental sustainability. The Sustainable Development Goal #2 is No Hunger, and #12 is Responsible Consumption and Production. Each of these can support and strengthen biotech decision making when the product supports a policy goal.

Many traits selected for integration into popular and commonly grown biotech varieties directly contribute to policies focused on food security and environmental sustainability. Potatoes with genetic resistance to late blight disease have protection that increases marketable yields, contributes to eradicating hunger in potato growing countries, and would reduce poverty in rural areas where farmers are affected by crop losses due to this disease. Similarly, biotech potatoes that produce more tubers on less land or decrease blackening due to polyphenol oxidase, would improve the sustainability of potato production by optimizing land usage and decreasing waste at harvest, processing, grocery stores, and homes. National development and sustainability policies offer a strong framework for regulatory agencies as they grapple with socio-political decisions for biotech varieties.

## Actionable recommendations

Assuming regulatory reform requires legislative rule making detracts from the many other implementation changes that can improve the efficiency of existing regulatory review processes and shorten the timelines for obtaining regulatory decisions. Our experience with obtaining approvals for biotech potato varieties in 12 countries suggests that three areas of regulatory implementation could have a positive effect on regulatory efficiency. The first is to ensure that data requirements are provided in guidance documents and are crop specific. This will help developers prepare submissions and reduce data collection and analysis by focusing on the safety of their crop variety. The second is to ensure that all reviewers are trained in risk assessment and the review process follows established risk assessment procedure. This will focus the review on questions important for safety and will filter out questions that might be of interest but have no relevance to safety. Part of achieving this risk assessment efficiency is to ensure that crop experts are available to the review committees and that there is good coordination between agencies to reduce duplication. The third area is to base regulatory decisions on the risk assessment outcome: i.e., the product is or is not safe for the local population. Where decisions include socio-political considerations, linking the product to national and locally adopted international policies provides a strong platform for decision making.

Regulatory efficiency is important for the release of new biotech varieties. Without a clear and efficient regulatory review and decision process, both public and private sector products will not be available to help improve farming livelihoods, food security, and environmental sustainability.
